# Morphology and Anatomical Classification of Pericardial Cavities: Oblique and Transverse Sinuses

**DOI:** 10.3390/jcm12134320

**Published:** 2023-06-27

**Authors:** Marian Burysz, Jakub Batko, Wojciech Olejek, Michał Piotrowski, Radosław Litwinowicz, Artur Słomka, Mariusz Kowalewski, Piotr Suwalski, Krzysztof Bartuś, Daniel Rams

**Affiliations:** 1 Department of Cardiac Surgery, Regional Specialist Hospital, 86-300 Grudziądz, Poland; 2 Thoracic Research Centre, Collegium Medicum Nicolaus Copernicus University, Innovative Medical Forum, 85-094 Bydgoszcz, Poland; 3 CAROL—Cardiothoracic Anatomy Research Operative Lab, Department of Cardiovascular Surgery and Transplantology, Institute of Cardiology, Jagiellonian University Medical College, 31-008 Krakow, Poland; 4 Department of Cardiovascular Surgery and Transplantology, Institute of Cardiology, Jagiellonian University Medical College, 31-008 Krakow, Poland; 5 Department of Pathophysiology, Ludwik Rydygier Collegium Medicum in Bydgoszcz, Nicolaus Copernicus University in Toruń, 85-094 Bydgoszcz, Poland; 6 Department of Cardiac Surgery and Transplantology, National Medical Institute of the Ministry of Interior and Administration, Wołoska 137 Str, 02-507 Warsaw, Poland; 7 Cardio-Thoracic Surgery Department, Heart and Vascular Centre, Maastricht University Medical Centre, Cardiovascular Research Institute Maastricht (CARIM), 6229 HX Maastricht, The Netherlands

**Keywords:** pericardial cavities, transverse sinus, oblique sinus, anatomy, video-assisted thoracoscopic procedures, VATS, minimally invasive surgery for atrial fibrillation, MIAFS

## Abstract

The pericardial sinuses are an important anatomical feature of the pericardial cavity, however, their clinical anatomy has not been thoroughly studied. In this study, we aim to provide the first classification of the oblique and transverse sinuses. We analyzed 121 computer tomography scans (46.3% female, age of 66 ± 12 years) of the pericardial cavity. The oblique sinuses were classified into four types: 1 (shallow with narrow entrance), 2 (shallow with wide entrance), 3 (deep with narrow entrance), and 4 (deep with wide entrance). The transverse sinuses were classified into four types: Concave, Wine-type, Straight, and Convex. The most common oblique sinus type was Type 1. The median oblique sinus volume was 8.4 (5.3) mL, the median entrance length was 33.0 (13.2) mm, and the depth was 38.2 (11.8) mm. The most common transverse sinus type was Concave. The median transverse sinus volume was 14.8 (6.5) mL, and the median length was 52.8 (17.7) mm. Our study provides an anatomical classification of the pericardial sinuses. The individual variability of the sinuses’ morphology highlights the importance of understanding the clinical topography of the sinuses, particularly for minimally invasive thoracic ablation procedures.

## 1. Introduction

### 1.1. Minimally Invasive Surgical Procedures Challenges

Surgical procedures involving the mediastinum require a comprehensive understanding of the anatomy to ensure safe and successful outcomes. With the increasing popularity of video-assisted thoracoscopic procedures (VATS), a high level of surgical skill and spatial orientation is necessary [1]. In addition, knowledge of the anatomy of the pericardial sinus is critical for minimally invasive surgery for atrial fibrillation (MIAFS) [2].

### 1.2. Anatomy of Pericardial Sinuses

The pericardial sinuses are passages within the pericardial cavity. Their size, volume, and shape vary individually [3]. Despite the clinical importance of the pericardial sinuses, the clinical anatomy of these structures has never been studied. The transverse sinus (TS) is a canal-shaped passage and its boundaries are defined by the nearby cardiac structures: pulmonary trunk and left atrial appendage limiting the left (arterial) entrance, superior vena cava and the right atrial appendage limiting the right (venal) entrance, superiorly by the inferior wall of the pulmonary trunk and pulmonary arteries, inferiorly by the atria and ventricles, anteriorly by the aorta, pulmonary trunk, and right ventricular outflow track. The oblique sinus (OS) is a J-shaped space, located, in most cases, posteriorly to the posterior wall of the left atrium. Its superior part is limited by the right and left pulmonary veins. Its right part is additionally bounded by the inferior vena cava. The inferior border of the OS is located superiorly to the great cardiac vein or coronary sinus. There has been no precise morphometric description of those structures performed to date.

### 1.3. Aim of the Study

Therefore, the aim of this study is to provide the first anatomical classification of the oblique and transverse sinuses based on their morphology, size, and volume.

## 2. Materials and Methods

### 2.1. Study Population

The study population consisted of 121 patients with long-term persistent atrial fibrillation (AF) who underwent contrast-enhanced electrocardiogram-guided computed tomography angiography scans between January 2016 and November 2018 for assessment of cardiothoracic topography and structural changes. The patients were 46.3% female and had a mean age of 66 ± 12 years. Depending on physician recommendation, 10 or 40 mg of propranolol or 40 mg of verapamil was administered to patients with a heart rate of over 70 bpm before the procedure. The imaging parameters for the CT were: 100–120 kV tube voltage and 350–400 mA effective tube current. During the imaging process, contrast medium at a dose of 1.0 ml/kg was injected at a rate of 5 mL/s, and the collimation was 2 × 32 × 0.6 mm and temporal resolution was 165 ms. In the test bolus, the acquisitions delay was the time of maximum density of the ascending aorta with an additional 6 s of delay. The images were reconstructed with a B26f and B46f kernel and an image matrix of 512 × 512 pixels. The 70% phase of multiphasic reconstruction (10 to 100%) was evaluated as the left ventricular end-diastolic phase and further studied.

### 2.2. Image Processing and Analysis

The image processing and analysis involved the semiautomatic segmentation of the left atrium (LA), right atrium (RA), left ventricle (LV), right ventricle (RV), pulmonary veins, coronary sinus, superior vena cava, inferior vena cava, coronary arteries, pulmonary trunk, aorta, TS, and OS at the predefined end-diastolic phase. The volumes of the LA, RA, LV, RV, TS and OS, entrance and depth of OS, TS length, height of venous and arterial entrance, and height of the middle of the TS were measured using virtual calipers in the Mimics Innovation Suite 23.0 visualization and three-dimensional reconstruction software (Materialise, Plymouth, MI, USA). The volume ratios were calculated as a consequence.

### 2.3. Definitions

The definitions used in the study included the entrance of the OS, which was defined as the shortest distance between the origins of the pulmonary veins, and the depth of the OS, which was defined as the distance from the upper OS border to the lower OS border, located near the sulcus coronarius. The TS venous entrance was defined as the end of the TS canal near the right atrial appendage and superior vena cava, while the TS arterial entrance was defined as the end of the TS channel located above the left atrial appendage and below the pulmonary artery. The TS length was defined as the distance from the center of the TS arterial entrance to the center of the TS venous entrance. The study also measured the TS venous entrance height as a distance from the lowest to the highest located point within the TS venous entrance, the TS arterial entrance height as a distance from the lowest to the highest located point within the TS arterial entrance, and the center height of the TS as a distance from the lowest to the highest located point within the half of the TS length.

### 2.4. Oblique Sinus Types

The study categorized the OS sinuses into four types based on their characteristics, including Type 1: shallow OS with narrow entrance, Type 2: shallow OS with wide entrance, Type 3: deep OS with narrow entrance, and Type 4: deep OS with wide entrance.

### 2.5. Transverse Sinus Types

The TS sinuses were also divided into four types, based on their entrance heights and middle height and their relations, including Type 1 (concave), Type 2 (wine-type) with subtypes 2a (wine bottle) and 2b (wine glass), Type 3 (straight), and Type 4 (convex).

### 2.6. Statistical Analyses

Descriptive statistics were used to present the obtained measurements. The Kruskal-Wallis test, Mann-Whitney U test, and chi-square test were used to determine whether there were any statistically significant differences in the measurements. The results were presented as the median and interquartile range (IQR), which was defined as the difference between the 75th and 25th percentiles of the data, in a Median (IQR) format. The statistical analyses were performed using the STATISTICA 13.3 software for Windows (StatSoft Inc., Tulsa, OK, USA).

## 3. Results

### 3.1. OS Morphometry

The median volume of the OS was 8.4 (5.3) mL. The median length of the OS entry was 33.0 (13.2) mm, and the depth was 38.2 (11.8) mm. The median entrance/height ratio of the OS was 0.9 (0.3), while the OS/LA volume ratio and the OS/atrial volume ratio were 5.9 (2.9)% and 3.1 (1.7)%, respectively.

### 3.2. OS Types

**Figure 1:**
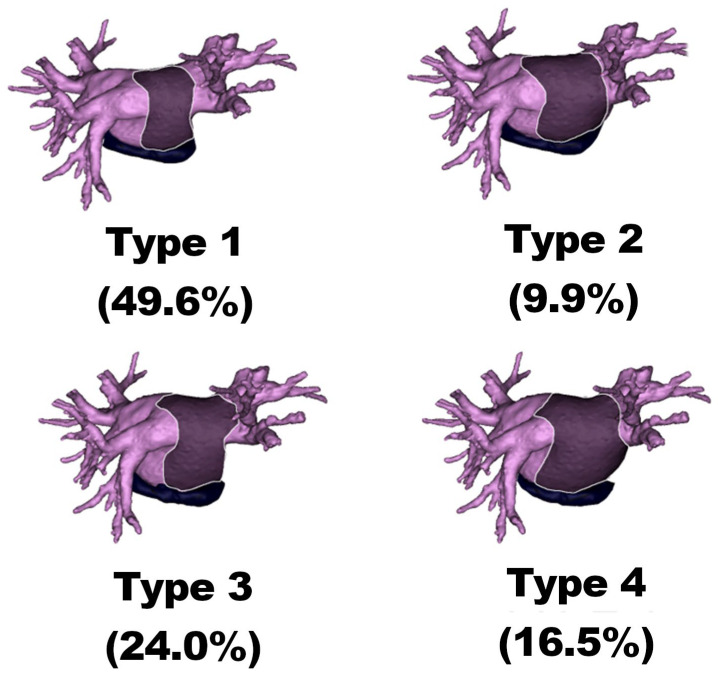
The oblique sinus types visual reconstruction and their prevalence.

The most common OS type was Type 1 (49.6%), while the least common was Type 2 (9.9%). Types 3 and 4 occurred in 24.0% and 16.5% of cases, respectively (see Figure 1).

#### 3.2.1. Type 1

The median volume of OS Type 1 was 6.9 (3.7) mL. The median length of the OS entry was 29.4 (9.0) mm, and the depth was 33.2 (5.0) mm. The median entrance/height ratio of the OS was 0.9 (0.3), while the OS/LA volume ratio and the OS/atrial volume ratio were 5.8 (3.1)% and 2.9 (2.0)%, respectively.

#### 3.2.2. Type 2

The median volume of OS Type 2 was 9.6 (6.6) mL. The median length of the OS entry was 45.7 (3.9) mm, and the depth was 36.2 (5.2) mm. The median entrance/height ratio of the OS was 1.3 (0.3), while the OS/LA volume ratio and the OS/atrial volume ratio were 7.5 (3.9)% and 3.4 (1.5)%, respectively.

#### 3.2.3. Type 3

The median volume of OS Type 3 was 8.2 (4.5) mL. The median length of the OS entry was 31.8 (7.9) mm, and the depth was 44.5 (5.9) mm. The median entrance/height ratio of the OS was 0.7 (0.3), while the OS/LA volume ratio and the OS/atrial volume ratio were 5.3 (1.8)% and 2.8 (1.3)%, respectively.

#### 3.2.4. Type 4

The median volume of OS Type 4 was 13.6 (6.0) mL. The median length of the OS entry was 47.5 (8.9) mm, and the depth was 52.1 (13.4) mm. The median entrance/height ratio of the OS was 0.9 (0.2), while the OS/LA volume ratio and the OS/atrial volume ratio were 6.8 (2.2)% and 3.6 (1.2)%, respectively.

### 3.3. Statistical Differences

There were statistically significant differences (*p* < 0.05) observed between the types in all measurements, except for the OS/atrial volume and OS/LA volume ratios. The OS volume was found to be the largest in Type 4 and the smallest in Type 1. The OS entry length was observed to be the greatest in Type 4 and the smallest in Type 1. The OS depth was greatest for Type 4 and smallest for Type 1. The OS entrance/height ratio was found to be the largest for Type 2 and the smallest for Type 3. The OS/LA volume ratio was greatest in Type 2 and smallest in Type 3. The OS/atrial volume ratio was found to be highest in Type 4 and lowest in Type 3. A summary of the measurements for each OS type is provided in Table 1.

### 3.4. TS Morphometry

The morphometric measurements of the TS are presented in this section. The median TS volume was 14.8 (6.5) mL, while the median TS length was 52.8 (17.7) mm. The median TS venous height and arterial entry height were 12.8 (4.3) mm and 12.1 (4.4) mm, respectively. The median TS center height was 8.3 (6.2) mm. The TS/atrial volume ratio was 5.0 (2.9)%.

### 3.5. TS Types

**Figure 2:**
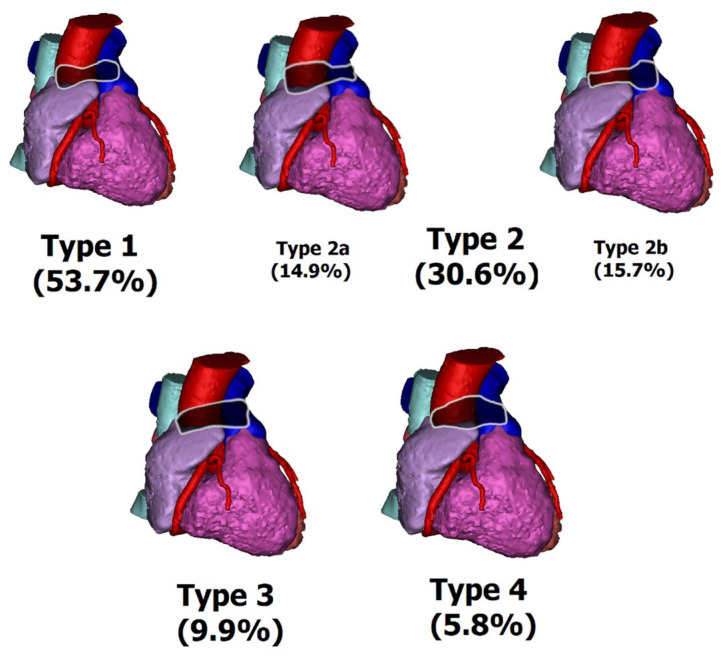
The transverse sinus types visual reconstruction and their prevalence.

Furthermore, the prevalence of the different TS types was also investigated. The most common TS type was Type 1, which was observed in 53.7% of cases, while the least common type was Type 4, with a prevalence of 5.8%. Type 2 (2a, 2b) and Type 3 were observed in 30.6% (14.9%, 15.7%) and 9.9% of cases, respectively (Figure 2).

#### 3.5.1. Type 1 (Concave Type)

The median volume of the TS was 14.7 (6.9) mL, with a median length of 52.9 (17.1) mm. The median TS venous height was 12.4 (4.0) mm, while the median arterial entrance height was 11.9 (4.0) mm. The median TS center height was 6.1 (2.4) mm, and the TS/atrial volume ratio was 5.3 (3.0)%.

#### 3.5.2. Type 2 (Wine Type)

For the wine type, the median TS volume was 15.1 (7.7) mL, with a median length of 50.6 (17.5) mm. The median TS venous height was 13.7 (4.9) mm, while the median arterial entrance height was 12.2 (5.5) mm. The median TS center height was 11.9 (5.2) mm, and the TS/atrial volume ratio was 5.4 (2.9)%.

#### 3.5.3. Type 2a (Wine Bottle Type)

The median TS volume for the wine bottle type was 16.5 (8.5) mL, with a median length of 48.7 (25.4) mm. The median TS venous height was 16.5 (2.8) mm, while the median arterial entrance height was 12.4 (7.8) mm. The median TS center height was 13.0 (4.1) mm, and the TS/atrial volume ratio was 5.6 (4.3)%.

#### 3.5.4. Type 2b (Wine Glass Type)

The wine glass type had a median volume of 14.6 (6.7) mL, with a median length of 54.5 (15.9) mm. The median TS venous height was 11.4 (3.7) mm, while the median arterial entrance height was 12.2 (5.6) mm. The median TS center height was 9.8 (5.7) mm, and the TS/atrial volume ratio was 4.8 (2.7)%.

#### 3.5.5. Type 3 (Straight Type)

The median TS volume for the straight type was 15.1 (11.5) mL, with a median length of 52.2 (19.6) mm. The median TS venous height was 13.6 (4.6) mm, while the median arterial entrance height was 11.9 (5.4) mm. The median TS center height was 13.1 (4.3) mm, and the TS/atrial volume ratio was 4.8 (2.2)%.

#### 3.5.6. Type 4 (Convex Type)

For the convex type, the median TS volume was 13.3 (6.8) mL, with a median length of 46.3 (24.2) mm. The median TS venous height was 9.3 (3.6) mm, while the median arterial entrance height was 11.4 (4.2) mm. The median TS center height was 21.0 (4.8) mm, and the TS/atrial volume ratio was 4.2 (2.2)%.

### 3.6. Statistical Differences

There were no statistically significant differences (*p* < 0.05) in the TS venous entrance heights and TS medial heights between the types. However, significant differences were observed in other parameters. The TS volume was greatest in Type 2a and smallest in Type 4. The TS length was the biggest in Type 2b and smallest in Type 4. The TS venous entrance height was greatest in Type 2a and smallest in Type 4. The TS arterial entrance height was greatest in Type 2a and smallest in Type 4. The height of the middle of the TS was highest in Type 4 and the smallest in Type 1. The highest TS/Atrial ratio was in Type 2a, and the lowest was in Type 4. Detailed comparison of the TS types can be found in Table 2.

## 4. Discussion

### 4.1. Atrial Fibrillation Management and Treatment

Atrial fibrillation is the most common cardiac arrhythmia, estimated to affect 1–2% of the adult population. AF and thromboembolic complications are independently associated with a 1.5- to 2-fold increased risk of all-cause mortality and morbidity [4]. Patient management consists of three main parts, abbreviated as ABC: A for anticoagulation and stroke prevention, B for symptom management, and C for cardiovascular and comorbidity optimization. In general, the CHA_2_DS_2_-VASc score scale is used to assess stroke risk and to implement anticoagulation treatment. In addition, the HAS-BLED score should be calculated to identify the possible risk of bleeding. For rhythm control, antiarrhythmic drugs should be used as the first choice and ablation as the second choice, especially if class I or class III antiarrhythmic drugs are not tolerated or cannot stabilize the patient’s heart rhythm. However, in selected patients, thoracoscopic or surgical ablation is recommended [4]. There are many methods for the endovascular treatment of AF, such as endocardial ablation or endovascular and hybrid left atrial appendage closure procedures for thromboprophylaxis [5,6,7]. However, when comparing the efficacy of AF treatments, surgical treatment is the most effective.

### 4.2. Minimally Invasive Atrial Fibrillation Treatment

In recent decades, MIAFS procedures have become established as an alternative to catheter ablation in the treatment of symptomatic paroxysmal or persistent AF with poor response to antiarrhythmic drugs or percutaneous AF ablation [4]. Additionally, this procedure can be used in patients with a low chance of successful catheter ablation. The reviews previously performed prove that MIAFS procedures have a significantly lower AF recurrence rate [8,9]. However, MIAFS procedures can be associated with a higher rate of immediate postprocedural complications than percutaneous treatment [8,9]. The goal of MIAFS is to perform a lesion set consisting of the isolation of the pulmonary vein atrium in combination with the isolation of the left atrial posterior wall (box). The complete electrical isolation of this area is critical for the restoration of the sinus rhythm. Furthermore, in MIAFS procedures, the simultaneous epicardial occlusion of the LAA is the gold standard, reducing both the local [10] and systemic prothrombotic status [11]. The thoracoscopic techniques currently on the market are mainly based on two types of ablation devices that use bipolar radiofrequency energy: Cardioblate Gemini-s (Medtronic, Minneapolis, MN, USA) [12] and EMR2 (Atri-Cure, Mason, OH, USA) [13]. Both techniques require the preparation of the transverse and oblique sinuses for epicardial ablation [14]. Therefore, knowledge of the topography of the transverse and oblique sinuses is a key element in the safety of the MIAFS procedure.

### 4.3. Video-Assisted Thoracoscopic Procedures

The technique of the VATS procedure, including visualization of the surgical field, differs from traditional open thoracic surgery. Although VATS procedures are more difficult to perform, the number of operations is systematically increasing because they offer advantages, such as less postoperative pain, faster recovery, lower risk of complications, and shorter hospital stays.

### 4.4. Lack of Anatomical Foundation for Minimally Invasive Procedures

Knowledge of the anatomy of the thorax and heart is one of the most important factors required to perform safe cardiac surgical procedures. The development of new techniques, especially those related to minimally invasive approaches, requires region-specific anatomic descriptions that are as detailed as possible. It is also critical to develop assessment techniques that can be further used in clinical situations. In recent years, clinical anatomy of the thorax and heart has evolved to provide information about anatomy-connected complications in cardiac surgery and serve as a basis for safer minimally invasive procedures [15,16].

Due to the year-to-year increasing number of research performed in the field of thoracoscopic AF ablation [17,18], there is a need for exploring additional factors that may lead to a decrease in the number of complications during the aforementioned procedure. A detailed description of the anatomy used in minimally invasive approaches can be found in Gelsomino et al. [2], who explains the mysteries of MIAFS anatomy in great detail [2]. In the above paper, we extended this topic to the anatomical knowledge of the shape and dimensions of the transverse and oblique cavities. The proposed classification can be used during minimally invasive procedures as a supporting factor for choosing the right individual anatomy-adapted surgical techniques.

### 4.5. Pericardial Sinuses Types Clinical Impact in MIAFS Procedures

Following Gelsomino et al. [2], the OS cavity is formed by the reflection of the serous pericardium around the pulmonary veins and vena cavae. The OS provides an expansion space for the MIAFS procedure because its anterior wall is formed by the posterior wall of the left atrium. In the physiological state, the volume of the oblique sinus is small, but in pathological conditions, such as cardiac tamponade, its volume can increase by a factor of a dozen.

Our study indicates that the average physiological volume of the OS is 8.4 mL, with a narrow entrance of 33 mm and a depth of about 38 mm. The most common morphological type is Type 1, which has a shallow cavity and narrow entrance. Type 2, which is reported to be the least common, differs from the previous type primarily in having a wider entrance, which provides the surgeon with better surgical options, especially when accessing the inferior pulmonary veins, and may be an additional factor promoting certain approaches in preoperative planning. Type 3, observed in nearly a quarter of the population, provides deeper access to the sinus, with the limitation of narrow access. From a surgical perspective, the most favorable type of OS is type 4, which has a deep cavity and wide entrance. It provides better access to the pulmonary veins and space for instruments during surgery. While OS seems to be a safer option compared to TS, due to its limited space for surgical instruments, special care should be taken to avoid damaging the surrounding veins.

On the other hand, TS is a tunnel-shaped passage from the left side to the right side of the pericardial cavity, with an average volume of 15 mL and a median length of 53 mm. The most common morphologic type is Type 1, which resembles a concave, with a much lower height in the center of the sinus compared with the entrances. Clinically, it limits the potential surgical space inside the sinus. Type 2, which should be subdivided during assessment, consists of two main types: 2a—wine bottle and 2b—wine glass. Type 2a is larger and has a significantly larger venal entrance, allowing easier access. Type 2b is smaller, with a typically larger arterial entrance. Type 3—the straight type—has a similar height along its entire length. Finally, type 4—convex—has small entrances with a large volume inside. Although access can be more difficult, a larger surgical area is available. Type 2a, with a large vein entrance and short length, is best suited for MIAFS procedures. TS is adjacent to the great vessels, which makes careful dissection of the superior vena cava and gentle friction of the fat in the triangle formed by the right pulmonary artery, the superior vena cava, and the RA necessary to avoid bleeding from the great vessels or the atria. Bleeding from these areas can be difficult to control and may require a complete sternotomy. Preoperative knowledge about the type of TS and OS can serve as additional support for the final decision about the surgical approach in the individual patient’s case.

### 4.6. Future Research Direction

The following questions should be addressed in future research:

- Do the type of AF and prior treatment affect OS and TS types and morphometry?
- Are certain TS or OS types associated with more or less intra- and post-operative complications?
- Do TS or OS morphometry and types change throughout life?
- What comorbidities affect TS or OS morphometry?
- Does successful AF treatment affect TS and OS morphometry?
- Do clinical outcomes differ between TS and OS types?

Answering the questions presented may deepen the understanding of the impact of AF on patients and allow safer, individualized treatment for each patient based on a personalized assessment. A multicenter, standardized study based on a large patient population with different types of AF and contrast-enhanced computed tomography or magnetic resonance imaging may serve as the basis for conducting the study. The implementation of TS and OS classification may serve as an additional factor that can be readily analyzed in future systematic reviews to answer these questions.

## 5. Conclusions

In recent years, minimally invasive methods such as VATS and MIAFS have gained popularity in cardiac surgery. However, our study shows that the success of MIAFS and patient safety depend on the surgeon’s knowledge of cardiac anatomy and topography. While surgical ablation is more effective than endocardial ablation in relieving atrial arrhythmias, the thoracoscopic approach has a higher rate of immediate postprocedural complications than percutaneous treatment. Therefore, ensuring the safety of patients is paramount when performing surgical procedures. This study proposes the first anatomical classification of the oblique and transverse sinuses, with precise morphometrical descriptions. Proper preoperative patient evaluation with the use of three dimensional reconstruction and anatomical analysis can positively influence patients’ treatment outcomes and lower complication rates due to a better understanding of patients’ individual anatomical characteristics.

## Figures and Tables

**Table 1:** Descriptive characteristics of oblique sinus classification. LA—left atrium, OS—oblique sinus, IQR—interquartile range.

	OS Classification								General (*n* = 121)	
	Type 1 (*n* = 60)		Type 2 (*n* = 12)		Type 3 (*n* = 29)		Type 4 (*n* = 20)			
	Median	IQR	Median	IQR	Median	IQR	Median	IQR	Median	IQR
OS volume [mL]	6.9	3.7	9.6	6.6	8.2	4.5	13.6	6.0	8.4	5.3
OS entry [mm]	29.4	9.0	45.7	3.9	31.8	7.9	47.5	8.9	33.0	13.2
OS depth [mm]	33.2	5.0	36.2	5.2	44.5	5.9	52.1	13.4	38.2	11.8
OS entry/height ratio	0.9	0.3	1.3	0.3	0.7	0.3	0.9	0.2	0.9	0.3
OS/Atrial volume ratio [%]	2.9	2.0	3.4	1.5	2.8	1.3	3.6	1.2	3.1	1.7
OS/LA volume ratio [%]	5.8	3.1	7.5	3.9	5.3	1.8	6.8	2.2	5.9	2.9

**Table 2:** Descriptive characteristics of transverse sinus classification. LA—left atrium, TS—transverse sinus, IQR—interquartile range.

	TS Classification												General (*n* = 121)	
	Type I (*n* = 65)		Type II (*n* = 37)		Type IIa (*n* = 18)		Type IIb (*n* = 19)		Type III (*n* = 12)		Type IV (*n* = 7)			
	Median	IQR	Median	IQR	Median	IQR	Median	IQR	Median	IQR	Median	IQR	Median	IQR
TS volume [mL]	14.7	6.9	15.1	7.7	16.5	8.5	14.6	6.7	15.1	11.5	13.3	6.8	14.8	6.5
TS length [mm]	52.9	17.1	50.6	17.5	48.7	25.4	54.5	15.9	52.2	19.6	46.3	24.2	52.8	17.7
TS venous entry height [mm]	12.4	4.0	13.7	4.9	16.5	2.8	11.4	3.7	13.6	4.6	9.3	3.6	12.8	4.3
TS middle height [mm]	6.1	2.4	11.9	5.2	13.0	4.1	9.8	5.7	13.1	4.3	21.0	4.8	8.3	6.2
TS arterial height [mm]	11.9	4.0	12.2	5.5	12.4	7.8	12.2	5.6	11.9	5.4	11.4	4.2	12.1	4.4
TS/atrial volume ratio [%]	5.3	3.0	5.4	2.9	5.6	4.3	4.8	2.7	4.8	2.2	4.2	2.2	5.0	2.9

## Data Availability

The data from the study are available upon reasonable request from the corresponding author.
